# Efficacy of raman spectroscopy in the diagnosis of kidney cancer: A systematic review and meta-analysis

**DOI:** 10.1097/MD.0000000000020933

**Published:** 2020-07-02

**Authors:** Hongyu Jin, Xiao He, Hui Zhou, Man Zhang, Qingqing Tang, Lede Lin, Jianqi Hao, Rui Zeng

**Affiliations:** a Department of Liver Surgery and Liver Transplantation Center, West China Hospital; b West China Clinical Skills Training Center, West China School of Medicine, Sichuan University; c Chengdu Women's and Children's Central Hospital, School of Medicine, University of Electronic Science and Technology; d West China School of Medicine; e Department of Cardiology, West China Hospital, Sichuan University, Chengdu, China.

**Keywords:** kidney cancer, raman spectroscopy, sensitivity, specificity

## Abstract

**Objective:**

To comprehensively analyze the relative effectiveness of Raman spectroscopy (RS) in the diagnosis of suspected kidney cancer.

**Patients and methods:**

We performed a complete systematic review based on studies from PubMed/Medline, EMBASE, Web of Science, Ovid, Web of Knowledge, Cochrane Library and China National Knowledge Infrastructure. We identified 2413 spectra with strict criteria in 6 individual studies published between January 2008 and November 2018 in accordance to Preferred Reporting Items for Systematic Reviews and Meta-Analysis guidelines. We summarized the test performance using random effects models.

**Results:**

General pooled diagnostic sensitivity and specificity of RS to kidney cancer were 0.96 (95% confidence interval [CI] 0.95–0.97) and 0.91 (95% CI 0.89–0.92). The pooled positive likelihood ratio (LR) was 9.57 (95% CI 5.73–15.46) while the negative LR was 0.04 (95% CI 0.02–0.11). The pooled diagnostic odds ratio was 238.06 (95% CI 77.79–728.54). The area under curve of summary receiver operator characteristics was 0.9466.

**Conclusion:**

Through this meta-analysis, we found a promisingly high sensitivity and specificity of RS in the diagnosis of suspected kidney masses and tumors. Other parameters like positive LR, negative LR, diagnostic odds ratio and area under curve of the summary receiver operator characteristics curve all helped to illustrate the high efficacy of RS in the diagnosis of kidney cancer.

## 1 Introduction

Kidney cancer including renal cell carcinoma (RCC) and transitional cell carcinoma occupies approximately 2% of all cancers worldwide. Unfortunately, a 2% to 3% increasing of mortality caused by kidney cancer has been observed and over 200,000 incidences and 100,000 deaths resulted from kidney cancer were reported each year.^[1]^ In order to enhance early diagnosis and treatment, more frequent use of imaging techniques was reported, especially abdominal computed tomography, adrenal glands computed tomography, which led to higher detection rate of renal masses.^[2]^ In patients with suspected renal masses who need long-term surveillance or prefer non-surgical interventions, renal mass biopsy (RMB) is often suggested to define the pathological features in order to provide guidance for subsequent treatment choices.^[3,4]^ However, a number of factors could have an impact on RMB diagnostic accuracy, including sampling errors, cut-off number of specimens and the interpretive degrees of pathologists.^[5]^ Accordingly, the diagnostic accuracy of RMB could be as low as 79%. Among these, important clinical diseases likely to be incorrectly identified included fibrosis and necrosis.^[6]^ Moreover, the appliance of RMB becomes even more limited when identifying tumor grade.^[7]^ Therefore, a more trustworthy diagnostic technique is urgently necessary.

In recent years, Raman spectroscopy (RS) has been applied clinically to either determine the benign and malignant essence of tumor or to identify the exact borderline between malignant and normal tissue during surgeries for its ability to optically characterize the internal compositional properties.^[8,9]^ Meanwhile, RS examination can be carried out in vivo and it's also real-time, label-free and nondestructive.^[10,11]^ Theoretically, RS detects variation of wave-lengths or Raman shifts resulted from the inelastic light scattering from certain molecules.^[12]^ Different molecules have distinct combinations of Raman shifts which can produce unique spectral signatures.^[13]^ Therefore, measured Raman spectra are highly related to the internal compositional features of tissues. Importantly, the availability to be performed in vivo and its label-free, real-time and non-destructive characteristics perfectly address the deficiencies of traditional RMB.

In the past decade, for the purpose to diagnose potential malignancies as early as possible, many clinical researches trying to confirm the diagnostic accuracy, sensitivity and specificity have been widely launched and a number of significant, meaningful outcomes have been generated. Couapel et al pointed out a 96% accuracy by RS in differentiating benign and malignant tumors in kidney.^[14]^ His team also found a precision rate of 80% and 96% in differentiating histological subtypes.^[15]^ Nevertheless, many of the studies concerning the accuracy, sensitivity and specificity of RS in defining unknown renal masses varied greatly from each other and several studies failed to recruit sufficient number of patient samples, which could have led to potential bias and inaccuracy. Thus, in order to comprehensively analyze the exact diagnostic efficiency of RS in determining the benign and malignant features of kidney tumors, we carried out this meta-analysis and systematic review in order to define the clinical value of RS.

## 2 Material and methods

### 2.1 Search strategy

Relying on the guidelines for performing meta-analysis, we searched widely acknowledged authenticated databases including PubMed/Medline, Web of Science, Cochrane Library, ClinicalTirals.gov (http://www.ClinicalTrials.gov), China National Knowledge Infrastructure for related articles published from January 2008 to November 2018. Articles we primarily searched and identified were subsequently screened for their quality, relevancy and availability. No language restriction was used. The keywords (query) of our primary search was as follows: ((((((((((kidney cancer) OR (renal cancer)) OR (renal cell carcinoma)) OR (RCC)) OR (kidney neoplasm)) OR (renal carcinoma)) AND (Raman spectroscopy)) OR (RS)) OR (efficacy)) OR (sensitivity)) OR (specificity).

### 2.2 Article selection

Two independent reviewers participated in the screening process to analyze the full texts and to perform quality assessments and relevancy determination. The main inclusion criteria included:

(1) reporting the use of RS in kidney cancers, including RCC and transitional cell carcinoma;
(2) being a randomized controlled trial and/or using any observational designs, including cross-sectional, case-control and cohort designs;
(3) reporting the sensitivity, specificity values or true positive (TP), false positive, true negative (TN) and false negative values, based on which sensitivity and specificity values could be calculated.

Meanwhile, we particularly excluded studies which were letters, editorials, case reports, and so on. Subsequently, we performed a blinded cross-check to detect underlying discrepancies. If a potential discrepancy was detected, a blinded third reviewer was assigned to adjudicate the conflict. The identification, inclusion and exclusion of studies were performed according to Preferred Reporting Items for Systematic Reviews and Meta-Analysis guidelines.

### 2.3 Data extraction

Two experienced investigators independently analyzed the final defined articles for primary parameters which indicated the diagnostic efficiency and secondary parameters concerning the basic information of the article. During the process, unexpected discrepancies were carefully discussed and resolved. In general, a total of 9 important diagnostic efficiency related parameters were extracted, including diagnostic sensitivity, specificity, accuracy, TP, TN, false positive, false negative values as well as spectra values. In addition, secondary parameters which reflected the baseline characteristics of the articles including title, first author, nationality, department, ethnicity, study design, sex and median age of the patients and enrollment year were also carefully extracted.

### 2.4 Statistical analysis

Data were extracted on either an article or study level when possible to reconstruct a 2 × 2 table, which we depended on to calculate sensitivity, specificity, positive predictive value (PPV), negative predictive value (NPV), odds ratios (ORs) and diagnostic likelihood ratios (DLRs) with their 95% confidence intervals (CIs). The forest plots were generated to display sensitivity and specificity estimates using Meta-Disc version 1.4 (Clinical Biostatistics Unit). To summarize test performance, two methods for meta-analysis diagnostic accuracy test were used: the bivariate model and the hierarchical summary receiver operating characteristic model.^[16,17]^ We chose to use these methods to respect the binomial structure of diagnostic accuracy data, thus jointly summarizing paired measures simultaneously, for example, sensitivity and specificity or, positive and negative likelihood ratios (LRs). Meanwhile, as a random effects approach, the bivariate/ hierarchical summary receiver operating characteristics meta-analysis allowed pooling results in view of knowing that heterogeneity was commonplace across included studies due to different or implicit thresholds. The said approach was carried out by metandi (Meta-analysis of diagnostic accuracy using hierarchical logistic regression) command in STATA 14.2 (StataCorp).

Additionally, summary receiver operator characteristics (SROC) curves were generated to assess the relationship between sensitivity and specificity. Meanwhile, the area under curve (AUC) was simultaneously calculated to evaluate the overall performance of RS. An excellent diagnostic effect was defined when AUC value was between 0.9 and 1; good when AUC value was between 0.8 and 0.9; fair when AUC value was between 0.7 and 0.8; poor when AUC value was between 0.6 and 0.7. The diagnostic method failed when AUC was between 0.5 and 0.6.^[18]^ The SROC curved was made through Meta-Disc version 1.4 (Clinical Biostatistics Unit).

### 2.5 Quality assessment

Standard quality evaluation of the included studies was performed based on the Quadas-2 tool.^[19]^ Particularly, the risk of bias was obtained by RevMan 5.3 (The Cochrane Collaboration). The articles were evaluated in the following processes: sequence generation (selection bias), allocation concealment (selection bias), blinding of participants and personnel (performance bias), blinding of outcome assessment (detection bias), incomplete outcome data (attrition bias), selective reporting (reporting bias) and others.

### 2.6 Publication bias

Publication bias was evaluated through Deeks Funnel Plot Asymmetry Test (consider the existence of publication bias when *P* < .05). The Deeks Funnel Plot Asymmetry Test was conducted by Stata 14.2 (StataCorp).

## 3 Results

### 3.1 Literature selection and screening

**Figure 1:**
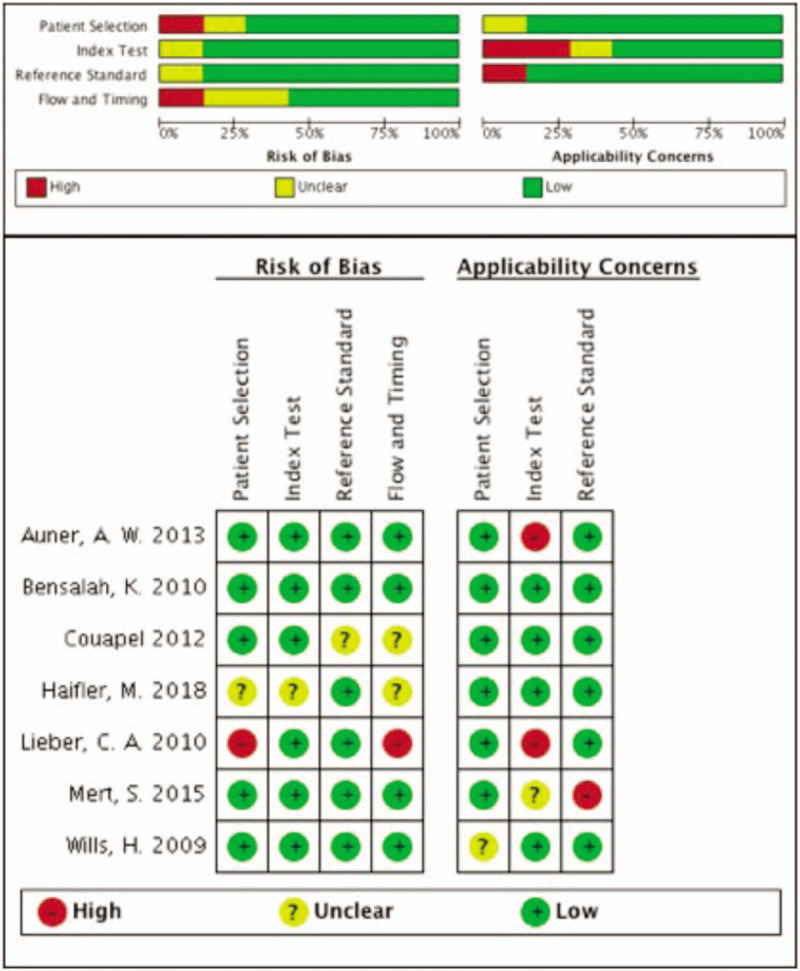
Flow diagram of literature search and selection process.

The initial database searching yielded 233 studies, among them 223 studies were obtained from authenticated databases, the other 10 articles were found through other sources. 37 studies were subsequently eliminated since they were obvious duplicates. After careful relevancy assessment, another 158 articles were further removed. Among the 40 studies left, 8 studies were not further evaluated since full-texts were unavailable (1 article) and were written in other languages (7 articles in total, among these 2 in Chinese, 2 in Japanese, 3 in German). Finally, only 7 articles were recommended for this meta-analysis after ones failing to meet the criteria mentioned above and case reports, reviews, editorials and letters were removed. The study selection and screening process is shown in Figure 1.

### 3.2 Characteristics of the included studies

After searching the authenticated databases, article screening and quality assessment process, 7 studies with high quality, reliability, comprehensible design and accessible full texts and data were considered for this systematic review. The total number of spectra from patients incorporated was 2413. In terms of the nationalities and regions, 4 studies were performed in North America (all in the United States), 3 in Europe (1 in Turkey, 2 in France). Specimens were collected from patients from January 2008 to November 2018. Diagnostic algorithm included discriminant function analysis in 2 articles, leave-1-specimen-out cross validation in 3 articles, local outlier factor in 1 article, principal components analysis in 1 article, linear discriminate analysis based on principal component analysis in 1 article and support vector machine in 1 article. Among the 7 recruited studies, 3 studies used Raman microscope, the other 3 studies used near-infrared RS and 1 study used Surface Enhanced Raman Scattering (SERS). In addition, 6 of 7 studies were carried out in vitro and 1 study in vivo. Spectra data were recorded in 5 articles. The detailed information of each study we included was shown in Table 1.

**Table 1:**
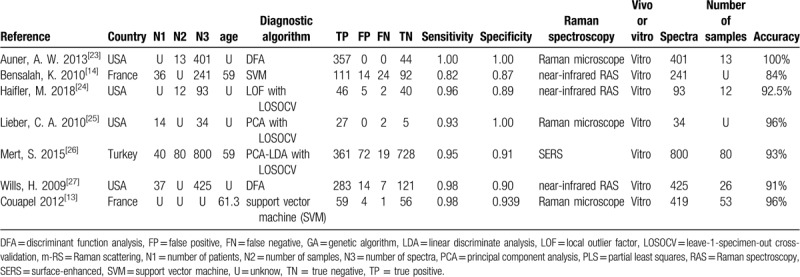
The basic information of the articles included.

### 3.3 Quality assessment of the included studies

**Figure 2:**
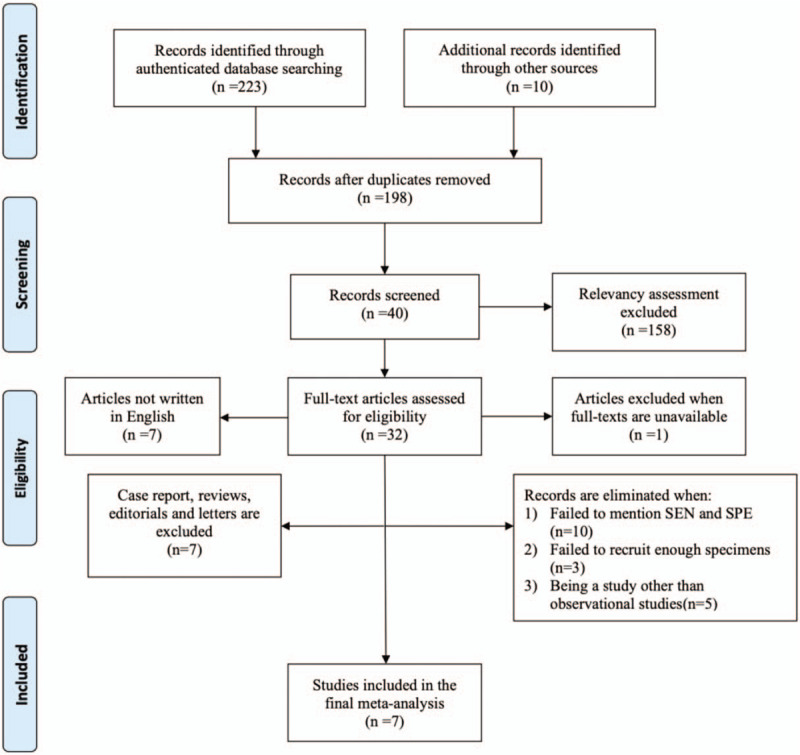
Quality assessment results by Quadas-2 tool. The risk of bias graph and the risk of bias summary.

Standard quality evaluation of the 7 included studies was performed based on the Quadas-2^[19,20]^ tool. According to the evaluating systems, the 7 included studies were ultimately defined as reliable (Fig. 2). However, the method used to select specimens may have contributed to bias.

### 3.4 Pooled results

Among the seven studies, 3 studies used RS to screen suspected kidney cancer with a purpose to tell apart the benign and malignant feature of a particular tissue. The other 3 studies used RS to explicit the borderline between benign and malignant tissues during a surgery. The additional 1 study provided the information of both the aspects. Since both the cancer screening process and borderline defining required the fundamental ability to tell the benign and malignant tissues apart, we recruited the two kinds of studies together to evaluate the general sensitivity and specificity. Still, we also did subgroup evaluation, in which studies concerning the screening efficacy were regarded as subgroup 1 and the studies investigating the borderline distinguishing ability of RS were regarded as subgroup 2.

#### 3.4.1 General pooled data

**Figure 3:**
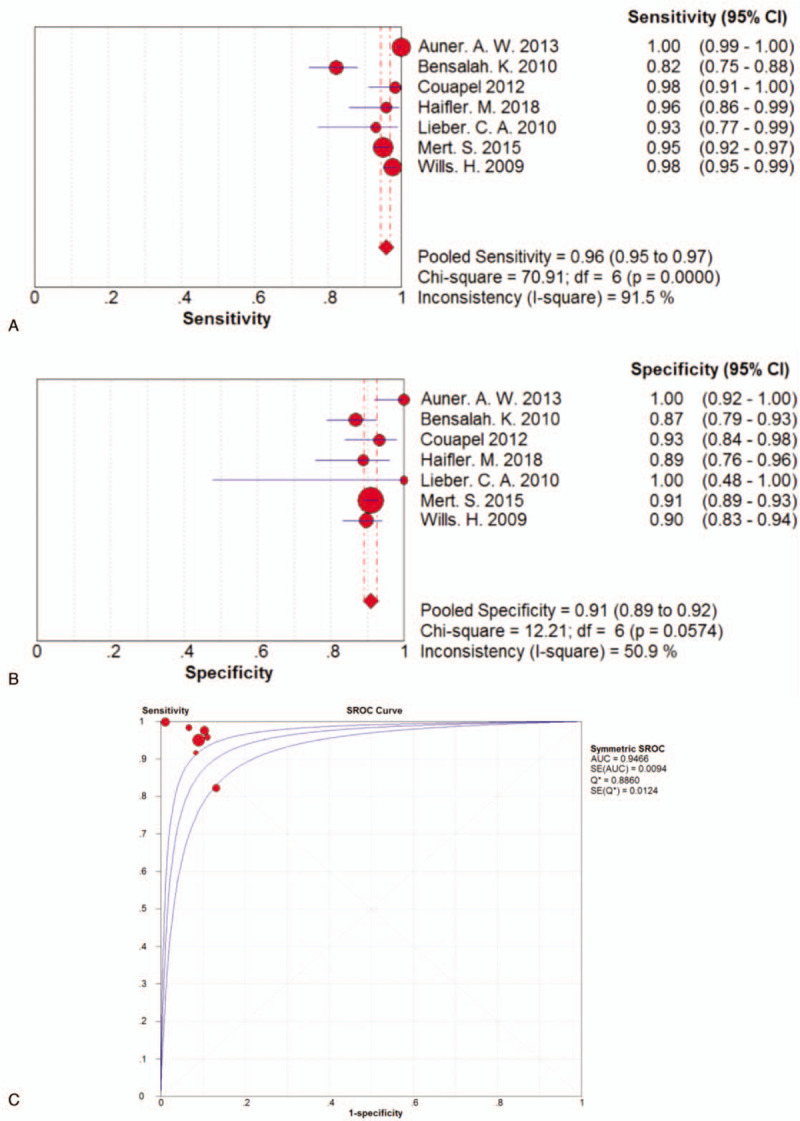
A. Forest plot of pooled sensitivity of the 7 studies included. B. Forest plot of pooled specificity of the 7 studies included. C. SROC curve of the 7 studies included.

The sensitivity of the 7 included articles ranged from 0.82 (95% confidence interval [CI] 0.75–0.88) in a study with 36 specimens (241 spectra) to 1.00 (95% CI 0.99–1.00) in a study which recruited 13 specimens (401 spectra). The pooled sensitivity was 0.96 (95% CI 0.95–0.97), which indicated a relatively low incidence rate of missed diagnosis. Particularly, among the 7 included studies, except for 1 with sensitivity of 0.82, the other 6 studies all maintained a sensitivity more than 0.90. The forest plots of pooled sensitivity of all the 7 studies were shown in Figure 3A.

The specificity of the 7 studies ranged from 0.87 (95% CI 0.79–0.93) in a study with 36 specimens (241 spectra) to 1.00 (95% CI 0.92–1.00) and 1.00 (95% CI 0.48–1.00) from studies with 13 (401 spectra) and 14 specimens (34 spectra) respectively. The general pooled specificity was 0.91 (95% CI 0.89–0.92), which was also a satisfactory parameter indicating a comparatively low rate of incorrect diagnosis. The forest plots of pooled specificity of all the 7 studies were shown in Figure 3B.

Among the recruited studies, positive LRs ranged from 8.63 (95% CI 3.77–19.75) to 89.87 (95% CI 6.71–1414.86) with a pooled positive LR of 9.57 (95%CI 5.73–15.46) by random effects model. Of all the studies, negative LR ranged between 0.00 (95% CI 0.00–0.02) and 0.20 (95% CI 0.14–0.30) with the pooled negative LR of 0.04 (95%CI 0.02–0.11) by random effects model. Based on positive and negative LRs, we pooled the diagnostic odds ratios (DORs) of the included studies and found the DORs ranged between 30.39 (95%CI 14.87–62.11) and 63635.00 (95%CI 1247.15–3246.9) with the pooled DOR of 238.06 (95% CI 77.79–728.54) by random effects model. The AUC was 0.9466. The SROC curve was displayed in Figure 3C.

#### 3.4.2 Pooled data of subgroup 1

**Figure 4:**
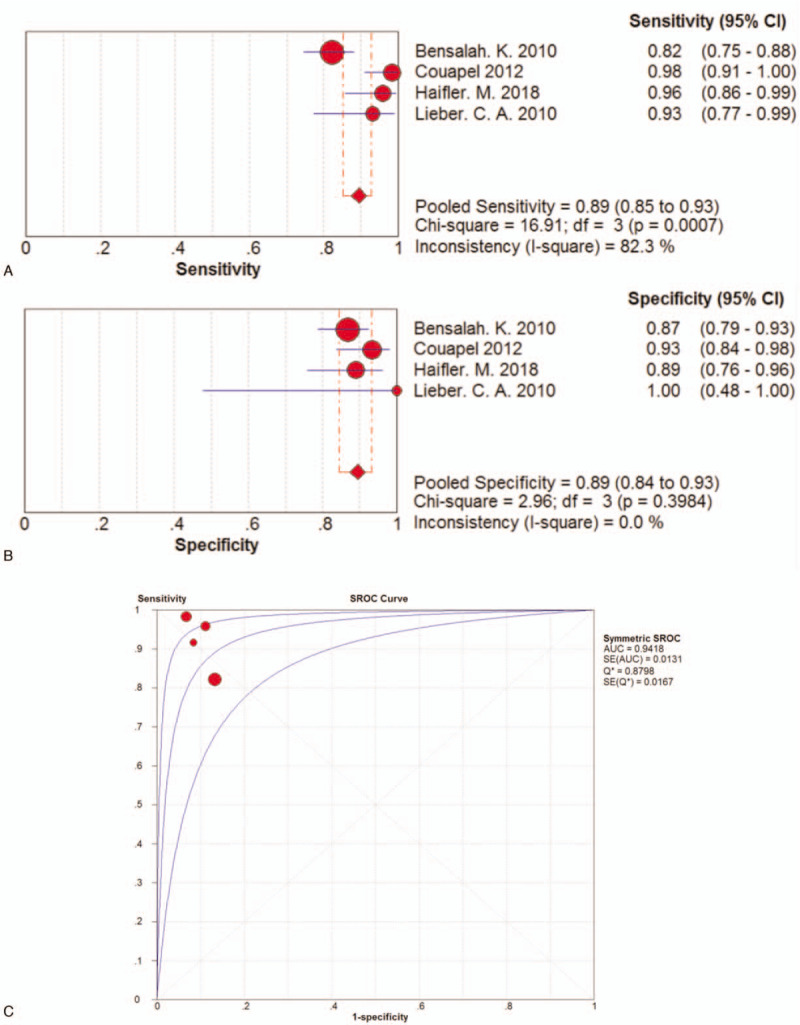
A. Forest plot of pooled sensitivity of the 4 studies in subgroup 1. B. Forest plot of pooled specificity of the 4 studies in subgroup 1. C. SROC curve of the 4 studies in subgroup 1.

The sensitivity of the 4 articles in subgroup 1 ranged from 0.82 (95% CI 0.75–0.88) to 0.98 (95% CI 0.91–1.00). The pooled sensitivity was 0.89 (95% CI 0.85–0.93), which was a little lower than sensitivity generated from all 7 studies, but still indicated quite a low incidence rate of missed diagnosis. Particularly, among the 4 included studies, except for 1 with sensitivity of 0.82, the other 3 studies all maintained a sensitivity more than 0.90. The forest plot of pooled sensitivity of RS used in kidney cancer screening process was shown in Figure 4A.

The specificity of the 4 studies concerning the identification of kidney cancer ranged from 0.87 (95% CI 0.79–0.93) to 1.00 (95% CI 0.48–1.00). The pooled specificity of the 4 studies was 0.89 (95% CI 0.84–0.93), which was also lower than the specificity generated from all 7 studies but was also a satisfactory parameter indicating a comparatively low rate of incorrect diagnosis. The forest plot of pooled specificity of all 7 studies was shown in Figure 4B. Among the 4 studies, positive LRs were between 6.23 (95% CI 3.80–10.21) and 14.75 (95% CI 5.72–38.04) with a pooled positive LR of 7.78 (95%CI 5.30–11.41) by random effects model. Of these studies, negative LR ranged between 0.02 (95% CI 0.00–0.12) and 0.20 (95% CI 0.14–0.30) with the pooled negative LR of 0.08 (95%CI 0.02–0.25) by random effects model. Of the 4 studies, ORs ranged between 30.39 (95%CI 14.87–62.11) and 826.00 (95%CI 89.57–7617.53) with the pooled diagnostic OR of 127.90 (95% CI 24.74–661.30) by random effects model. The AUC was 0.9418. The SROC curve was shown in Figure 4C.

#### 3.4.3 Pooled data of subgroup 2

**Figure 5:**
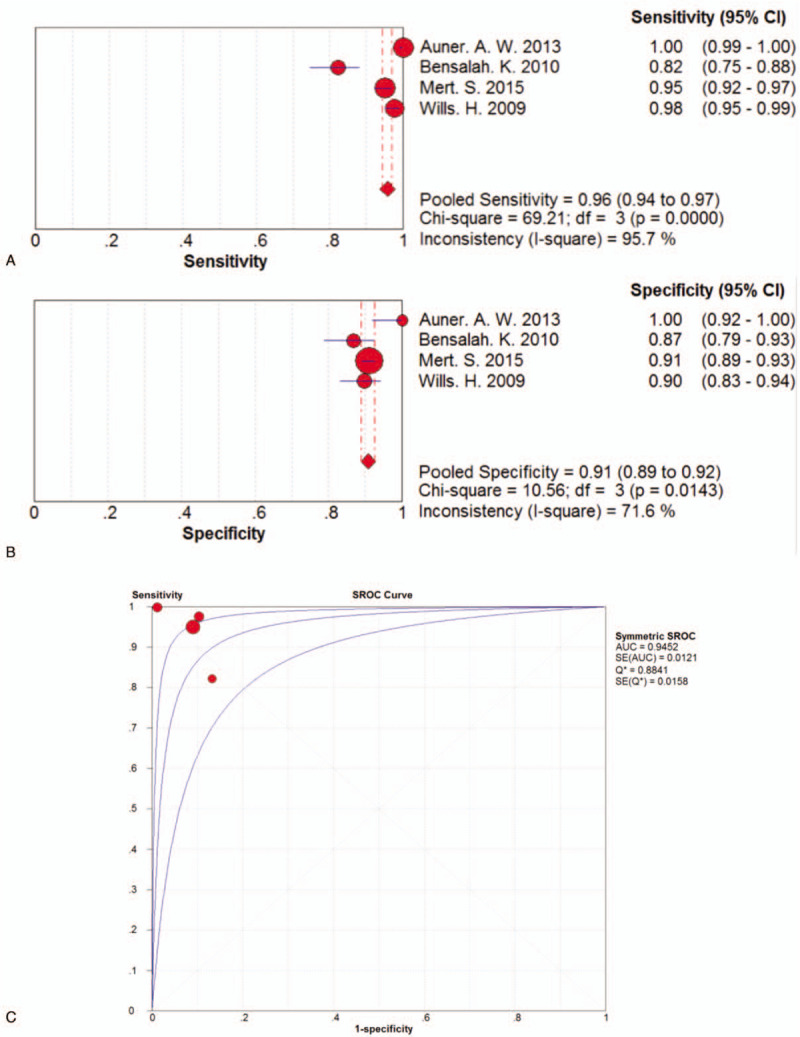
A. Forest plot of pooled sensitivity of the 4 studies in subgroup 2. B. Forest plot of pooled sensitivity of the 4 studies in subgroup 2. C. SROC curve of the 4 studies in subgroup 2.

The pooled sensitivity of subgroup 2 was 0.96 (95%CI 0.94–0.97) lying between 0.82 (95%CI 0.75–0.97) and 1.00 (95%CI 0.99–1.00). The pooled sensitivity in subgroup 2 was shown in Figure 5A. The pooled specificity of subgroup 2 was 0.91 (95%CI 0.89–0.92) ranging between 0.87 (95%CI 0.79–0.93) and 1.00 (95%CI 0.92–1.00). The pooled specificity of subgroup 2 was displayed in Figure 5B. The positive LR of subgroup 2 fluctuated between 6.23 (95%CI 3.80–10.21) and 89.67 (95%CI 5.71–1414.86) with a pooled positive LR of 9.21 (95%CI 6.32–13.40) by random effects model. Meanwhile, the pooled negative LR was 0.04 (95%CI 0.01–0.15) by random effects model, which ranged from 0.00 (95%CI 0.00–0.02) to 0.20 (95%CI 0.14–0.30). The DOR ranged from 30.39 (95%CI 14.87–62.11) and 63635.00 (95%CI 1247.15–3246931.96) with a pooled DOR of 235.01 (95%CI 52.18–1058.39). The AUC was 0.9452, which was shown in Figure 5C.

## 4 Discussion

This meta-analysis was performed according to the standard protocol for systematic reviews, involving a total of 7 articles, 2431 spectra into consideration. Two independent reviewers were assigned in study screening, data extraction and quality assessment process. Methodologies including heterogeneity exploration and SROC curve analysis were simultaneously applied.

RS is a novel, optical spectroscopic technique with the potential for in vivo tracking of overall biomolecular changes in cells and tissues. In particular, RS was shown as a promising tool to reveal biochemical and metabolic changes that occur in cancer progress at the cellular level. In this scenario, ccRCC is characterized by a reprogramming of energetic metabolism. In particular, the metabolic flux through glycolysis is partitioned, and mitochondrial bioenergetics and OxPhox are impaired.^[21–23]^

This systematic review and meta-analysis confirmed the superiority and high diagnostic efficacy of RS in the identification of kidney cancer. Through this meta-analysis, we found the general pooled diagnostic sensitivity and specificity of RS to kidney cancer were 0.96 (95% CI 0.95–0.97) and 0.91 (95% CI 0.89–0.92). Since a sensitivity and specificity over 0.90 were observed, a high efficacy of early diagnosis of suspected small renal lesion and mass was reconfirmed, with a comparatively low incidence of both missed and incorrect diagnosis. The diagnostic accuracy was reported in 4 studies, ranging from 0.84% and 100%, which were also satisfactory. Compared with traditional RMB, whose accuracy was noted fluctuating around 85%, RS diagnostics provided more confident and precise outcomes. Moreover, we found a general pooled DOR of 238.06 (95% CI 77.79–728.54) by random effects model, with the smallest DOR in a single study of 30.39 (95%CI 14.87–62.11). Since a DOR over 1 indicated a high discriminant effect and the discriminant effect increased with DOR value, the general DOR of RS to kidney cancer reflected a trustworthy diagnostic efficacy. In SROC curve analysis, AUC was 0.9418. According to the standard grading system for SROC, the diagnostic efficiency was regarded as excellent. However, despite the high sensitivity and specificity, we still need to be cautious when choosing examinations for a specific patient, for example RS is not necessarily better when trying to determine the histological type of the patient.

The diagnostic efficacy of RS to identify the benign and malignant feature of a certain tissue or to manifest the borderline between normal and malignant tissue were separately analyzed in subgroups. Pooled sensitivity and specificity for subgroup 1 were 0.89 (95% CI 0.85–0.93) and 0.89 (95% CI 0.84–0.93) respectively, in which a low rate of missed and wrong diagnosis was illustrated. In the meantime, the pooled sensitivity and specificity for subgroup 2 were also noted, which were 0.96 (95%CI 0.94–0.97) and 0.91 (95%CI 0.89–0.92). Therefore, a clear and promising borderline identification could be expected.

RS was primarily applied in the field of chemistry and was transformed into a medical diagnostic tool later in order to provide safe, convenient and trustworthy diagnostic methodology.^[24–26]^ In 1995, the first appliance of RS in the genitourinary tract was reported in bladder cancer, and gradually expanded to kidney and prostate in the 21^st^ century.^[27]^ In 2013, Auner A.W. documented a 100% accurate experiment of RS to kidney cancer, with sensitivity and specificity of 1.00.^[28]^ In this case, 13 patients and 401 spectra were analyzed with a TP and TN of 357 and 44 respective. Because this study did recruit sufficient number of samples and spectra, we supposed the extremely high accuracy, sensitivity and specificity were caused by the original high diagnostic efficiency. Meanwhile, we also found a study reporting a much lower sensitivity, specificity values compared with other studies which included 36 samples and 241 spectra.^[29,30]^ A Closer observation to the sample composition, especially tumor stage, tumor grade of this study indicated that the proportion of high-grade tumors and pt2, pt3 stages were significantly lower than those in other studies.^[31,32]^ Thus, this might have contributed to the fluctuation of parameters. Therefore, after eliminating related influencing bias, we confirmed the high diagnostic effectiveness manifested by sensitivity and specificity.

Through this study, we confirmed the ability of early diagnosis of RS in kidney cancer which would provide certain clinical benefits, since early-stage patients can be efficiently selected with the help of such diagnostic tool.^[33–36]^ Moreover, if RS can further offer more solid evidences on disease staging, it can make it easier for physicians to arrange a more well-rounded treatment plan.^[37]^

Still, we acknowledged several limitations in this study. Firstly, RS has not been widely admitted as a normal clinical diagnostic tool, therefore inadequate number of clinical researches were published, which absolutely lowered the number of articles we could include. Secondly, due to the small number of studies incorporated, we failed to calculate specific sensitivity and specificity of every subgrade of tumors, especially tumors of different pathological and clinical subtypes. Further comprehensive study can target the subtypes in order to provide more precise clues for clinical practice.

## 5 Conclusion

Through this meta-analysis, we found a promisingly high sensitivity and specificity of RS in the diagnosis of suspected kidney mass and tumors. Other parameters like positive, negative LR, DOR and AUC of the SROC curve all helped to illustrate the high efficacy of RS in the diagnosis of kidney cancer.

## Author contributions

**Conceptualization:** Hongyu Jin, Hui Zhou, Rui Zeng.

**Data analysis:** Hui Zhou, Man Zhang.

**Data collection or management:** Hongyu Jin, Xiao He.

**Data curation:** Hongyu Jin, Hui Zhou.

**Formal analysis:** Hongyu Jin, Lede Lin, Jianqi Hao.

**Funding acquisition:** Rui Zeng.

**Investigation:** Hongyu Jin, Hui Zhou, Man Zhang, Qingqing Tang, Jianqi Hao.

**Manuscript writing review and editing:** Hongyu Jin, Man Zhang, Rui Zeng.

**Methodology:** Hongyu Jin, Xiao He, Hui Zhou, Man Zhang, Qingqing Tang, Jianqi Hao

**Project administration:** Rui Zeng.

**Protocol/Project development:** Hongyu Jin, Jianqi Hao, Lede Lin.

**Software:** Hongyu Jin, Hui Zhou, Man Zhang.

**Validation:** Hongyu Jin.

**Writing – original draft:** Hongyu Jin, Xiao He.

**Writing – review & editing:** Hongyu Jin, Rui Zeng.
